# Passive appendages generate drift through symmetry breaking

**DOI:** 10.1038/ncomms6310

**Published:** 2014-10-30

**Authors:** U. Lācis, N. Brosse, F. Ingremeau, A. Mazzino, F. Lundell, H. Kellay, S. Bagheri

**Affiliations:** 1 Linné Flow Centre, KTH Royal Institute of Technology, Department of Mechanics, 10044 Stockholm, Sweden; 2 Department of Mechanical and Aerospace Engineering, Princeton University, Princeton, New Jersey 08544, USA; 3 Department of Chemical, Civil and Environmental Engineering (DICCA), University of Genova, 16145 Genova, Italy; 4 INFN and CINFAI Consortium, Genova Section, 16146 Genova, Italy; 5 Université de Bordeaux, Laboratoire Ondes et Matière d'Aquitaine (UMR 5798 CNRS), 351 cours de la Libération, 33405 Talence, France

## Abstract

Plants and animals use plumes, barbs, tails, feathers, hairs and fins to aid locomotion. Many of these appendages are not actively controlled, instead they have to interact passively with the surrounding fluid to generate motion. Here, we use theory, experiments and numerical simulations to show that an object with a protrusion in a separated flow drifts sideways by exploiting a symmetry-breaking instability similar to the instability of an inverted pendulum. Our model explains why the straight position of an appendage in a fluid flow is unstable and how it stabilizes either to the left or right of the incoming flow direction. It is plausible that organisms with appendages in a separated flow use this newly discovered mechanism for locomotion; examples include the drift of plumed seeds without wind and the passive reorientation of motile animals.

## Abstract (web summary)

Passive mechanisms without energy input are the only way for non-motile organisms to disperse in fluids. Here, the authors use the analogue of the inverted pendulum motion upon gravity to explain the passive drift of a body with a protrusion to the sides of an incoming fluid stream.

## Graphical abstract

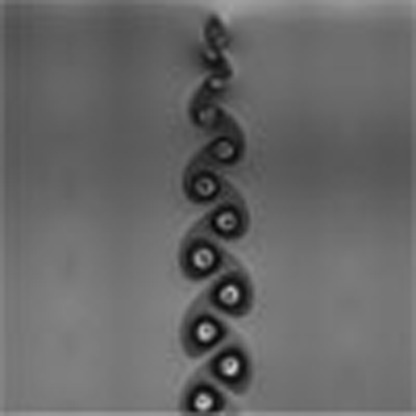

Aerial and aquatic animals have developed distinct and complex mechanisms to move through air and water with little resistance^1^,^2^. To efficiently manipulate the surrounding flow, they combine both active and passive methods^3^. By actively flapping, undulating or oscillating appendages, the animal generates forces that displace the surrounding fluid, which in turn pushes the animal in the desired direction. The contribution to locomotion through passive mechanisms, on the other hand, is much harder to identify. This is because the function of a non-smooth compliant skin^4^,^5^, hair, feathers and other passive protrusions are not only related to the movement of the animal, but also to other features, such as sensation, protection and insulation. The advantage of passive locomotion techniques is that no energy needs to be expended by the animal; instead the energy is extracted through a complex interaction with the environment. Forces are generated through instabilities that are often nucleated at the boundary of the fluid and elastic structures^6^,^7^. Passive mechanisms are also the only way for non-motile organisms to disperse.

When a body travels through a fluid at sufficiently high speed, there is a difference in pressure between the front and rear surfaces of the object. As explained by Prandtl’s boundary layer theory in 1905^8^, this pressure drag—which is often undesired for locomotion—is a consequence of flow separation (that is, a region where the flow becomes detached from the surface of the body and has the form of vortices and eddies). In this manuscript, we show that a separated region behind a body may in fact be exploited to aid locomotion. This becomes possible when an appendage of simple shape is added to the body. As we will show, the resting position of a short protuberance in a separated flow is unstable in the same way as an inverted pendulum is unstable under gravity. The protuberance stabilizes at an angle (up to 40 degrees) either to the left or right of its resting position and, as a consequence, a net drift/lift force (transverse to drag force) is generated. Although, not explicitly demonstrated in this paper, it is likely that this symmetry breaking has implications for locomotion^9^; the trailing part of a cephalopod shell^10^, the tail of a gliding tadpole^11^, the hind-wing tails of the swallowtail butterfly^12^ and the pop-up feathers of many birds^13^ are a few examples of appendages that are susceptible to this instability. We use experiments and numerical simulations to show the existence of an inverted-pendulum-like (IPL) instability under a wide range of conditions (steady/unsteady flows, rigid/elastic protrusions and fixed/falling objects) and that it generates rotation and drift of the body. We then unravel the mechanism with a simple model—which will justify the term IPL—that provides quantitative prediction of the induced rotation and drift.

## Results

### Experiments of a rigid plate attached to a cylinder

**Figure 1:**
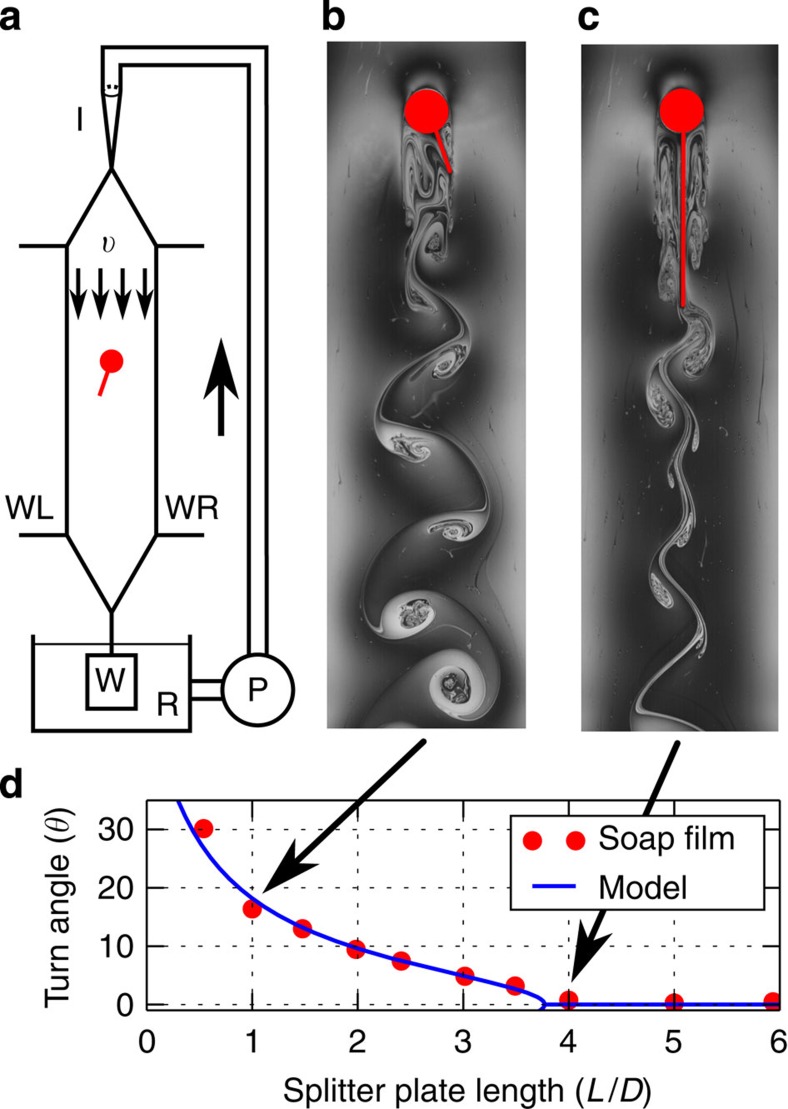
Soap-film experiments of a rigid plate attached to a cylinder. (**a**) Schematic of the soap-film apparatus. Wires WL and WR are thin nylon wires (diameter=1 mm). The film hangs from the wires. The bottom reservoir contains a soapy solution made with water and 2% of dishwashing detergent. A gear pump pumps the solution to the injection nozzle; after which it forms a flowing thin film between the wires before it returns to the reservoir (for details see Methods: Soap-film experiments of a fixed body). In **b** and **c** red colour depicts the body, which is free to rotate around the centre of cylinder. Grey contours are visualizations of the vortical structures in the flow provided by interference fringes in yellow light (wavelength≈589 nm). Whereas the vertical position is stable for the long plate (**c**), it is unstable for the short plate (**b**), which stabilizes at an skewed angle of 16 degrees. (**d**) Keeping the film velocity fixed at *υ*=1.87±0.05 m s^−1^, the average turn angle (in degrees) of a cylinder with a splitter plate of different lengths indicates a bifurcation at *L*_c_=(4.0±0.2)*D*. The s.d. of the splitter-plate position is close to one degree for all lengths. The prediction of the analytical model (*B*_max_=2.6*D*, *k*=1.0) is shown with a blue solid line.

To show evidence of the IPL instability, we carried out experiments in which a soap film^14^,^15^,^16^ flows vertically (due to gravity) at a constant velocity *υ*=1.9 m s^−1^ between two wires (Fig. 1a). Inside the soap film, we placed a circular cylinder of diameter *D*=6.3 mm with a clamped rigid splitter plate of length *L*. The body is free to rotate around the centre of the cylinder. When the plate is longer than the critical length of *L*_c_=(4.0±0.2)*D*, the body will—in the presence of any perturbation—always restore to a steady symmetric straight position (Fig. 1c). However, for a shorter plate (*L*=1.0*D*) the body stabilizes at an angle of 16 degrees to the right of the incoming stream (Fig. 1b). It is of equal probability that the plate settles to the right or left of the incoming stream of flow. In Fig. 1d, we show how the time-averaged turn angle *θ* depends on the splitter-plate length. A clear transition is observed from a symmetric state to an asymmetric one at a critical threshold.

**Figure 2:**
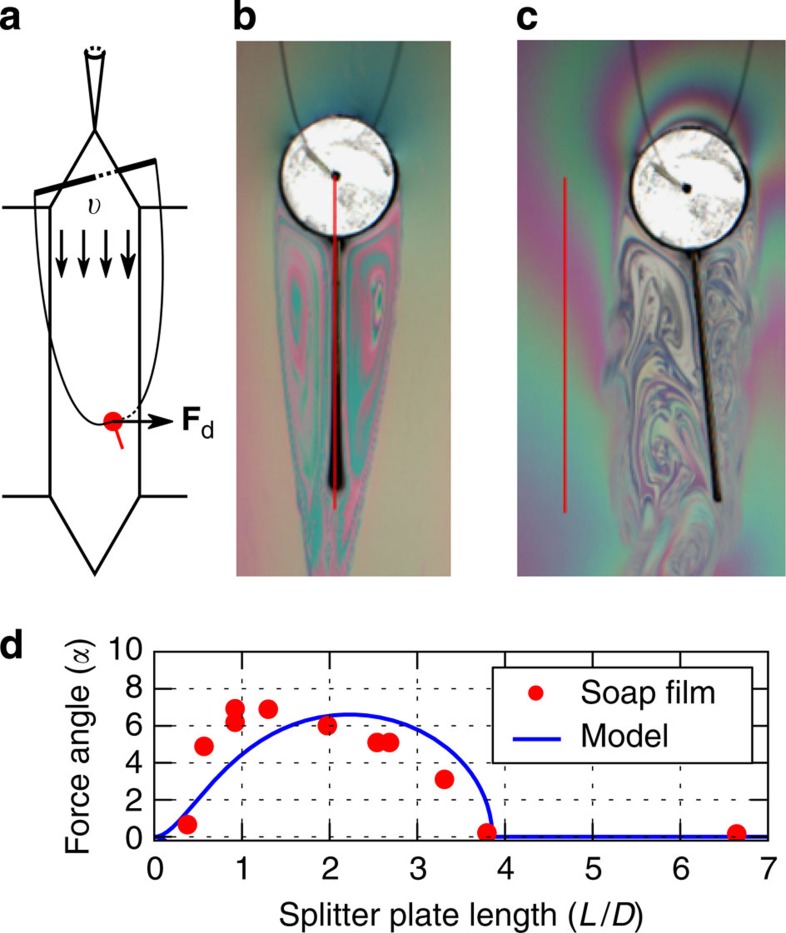
Soap-film experiments of a freely hanging body. (**a**) Schematic of the soap-film apparatus and the hanging mechanism for the cylinder with a splitter plate. The object is attached to a nylon wire at the cylinder centre. The nylon wire is then connected on both sides to a fixed rod. If the cylinder with a splitter plate experiences a drift force **F**_d_, the object drifts away from the centre position until it reaches equilibrium with gravitational force (see Methods: Soap-film experiments of a hanging body). (**b**) For a low flow velocity we observe a straight, symmetric position of the plate (depicted with red line). (**c**) For a high flow velocity, the object turns and drifts to the right. (**d**) Experimental observation of the force angle versus the length of the splitter for a velocity *υ*=2.0 m s^−1^ (red dots). Model predictions in blue with parameters *B*_max_=2.6*D* and *k*=1.0.

To show the existence of a drift force *F*_d_ on the body as a result of the symmetry breaking, we fixed the same object on a ‘loose’ pendulum made of a thin nylon wire (Fig. 2a). This wire crosses the film perpendicularly through a small hole drilled in the centre of the cylinder. If the object—which is free to rotate under the imposed conditions—turns, we expect a non-zero drift force on the object that will induce a displacement of the equilibrium position of the pendulum. Figure 2b,c shows snapshots of the cylinder with a splitter plate of length *L*=2.1*D* for low and high film velocities, respectively. We observe that when the flow velocity is high, the average turn angle *θ* is non-zero (asymmetric state) and the whole object has drifted sideways, resulting in a new equilibrium position of the pendulum (Supplementary Movies 1 and 2). Moreover, we observe from Fig. 2c that the disk drifts in the same direction as the splitter plate is tilted.

We calculate the corresponding drift angle^2^ *α*, which can be obtained from the ratio of the drift force to the drag force, that is,


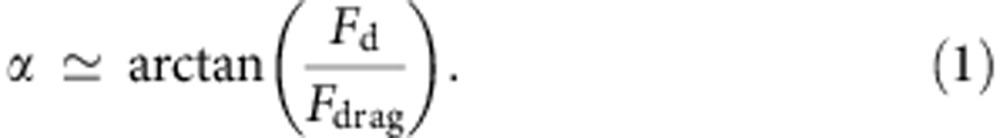


We estimated *F*_drag_ by fixing the cylinder to the end of a calibrated cantilever perpendicular to the soap-film plane and measuring its deflection (the procedure is depicted in Supplementary Fig. 1). We estimated the drift force *F*_d_ from a force-balance equation (see Methods: soap film experiments of hanging body). The average drift angle as a function of the splitter-plate length is shown in Fig. 2d. We observe that short plates that have a non-zero turn-angle (*θ*) also have a non-zero drift angle *α*, confirming the instability-induced forcing. Moreover, as we approach short and long appendage limits (*L*→0 and *L*→*∞*, respectively), the drift angle tends to zero. It is also observed that there exists an optimal value of splitter-plate length for maximum drift force. The existence of an optimal configuration may be an important factor in evolution of tails and appendages of motile animals, because it is often desirable to move in a specific direction as fast as possible.

### Simulations of a free-falling cylinder with a rigid plate

**Figure 3:**
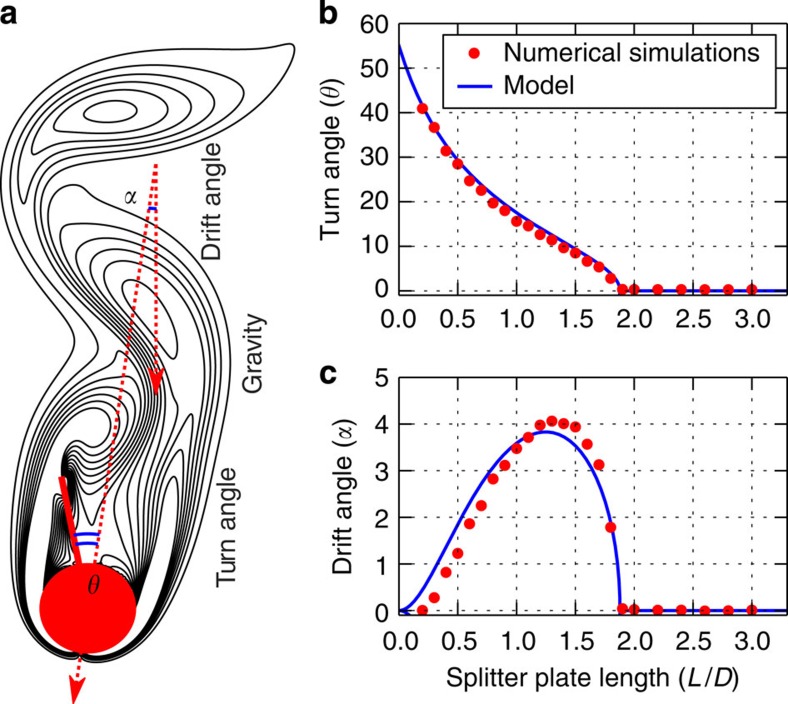
Numerical simulations of a free-falling cylinder with a splitter plate. The problem is defined by three dimensionless parameters; Reynolds number *Re*, solid and fluid density ratio *ρ*=*ρ*_s_/*ρ*_f_ and splitter-plate length ratio 
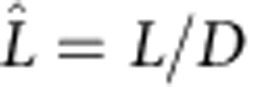
. (**a**) Vorticity contour levels (solid lines) show the von Kárman vortex street developing in the wake of the falling body (*Re*=156, *ρ*=1.01, 
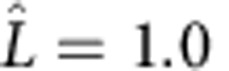
). As a result of the IPL instability, the body turns by *θ*=19 degrees and drifts by *α*=8 degrees with respect to gravity (Supplementary Movie 3). The turn and drift angles as functions of the splitter-plate length are shown, respectively, in **b** and **c** for *Re*=45 and *ρ*=1.001. Predictions of the analytical model (*B*_max_=1.26*D*, *k*=0.90) are shown with solid blue lines in **b** and **c**.

We complement our experiments with two-dimensional numerical simulations of a free-falling cylinder with a splitter plate clamped to its rear end. This allows us to investigate two orders of magnitude lower Reynolds numbers *Re*=*UD*/*ν* (*U* being the descent speed of the body, and ν the kinematic viscosity of the fluid) as well as to demonstrate how the instability generates a lateral motion. We found that when the body with a splitter plate shorter than a critical length is released, the body rotates an angle *θ* towards a new equilibrium, and drifts at a constant angle *α* with respect to the straight vertical path. Whereas *θ* is due to the symmetry-breaking-induced torque, the drift angle *α* is a manifestation of the induced transverse force. Figure 3a shows an instantaneous snapshot of the unsteady vorticity field forming behind a falling body (*Re*=156) during steady drift. A constant drift angle in an unsteady wake could not have been anticipated from fixed-body experiments, as it is well-known that freely falling bodies may have highly non-trivial descent paths due to wake-induced oscillations^17^.

Next, we show how the turn and drift angles depend on the splitter-plate length for a steady wake (*Re*=45). In Fig. 3b, a distinct bifurcation from a straight position of the plate (*θ*=0) to a skewed position (*θ*≠0) is observed. Figure 3c shows that plates with a non-zero turn angle have an oblique path (*α*≠0). The drift angle in Fig. 3c displays the same features as the drift angle from our soap-film experiments in Fig. 2d, namely, that as *L*→0 the drift angle tends to zero and that there exists an optimal length for maximum drift. Despite that the Reynolds number of the computations is several orders of magnitude smaller than the experiments (*Re*=12,000), we have a good qualitative agreement between the two for both turn and drift angles.

### A theoretical model

**Figure 4:**
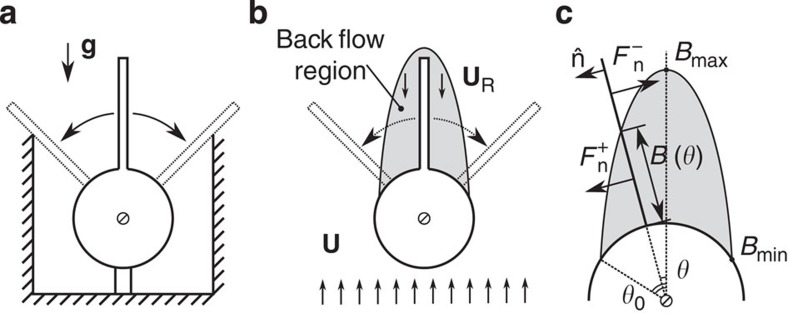
Model of IPL instability. (**a**) Schematic of an inverted pendulum consisting of a plate on top of a cylinder, which is free to rotate. Due to gravity, the symmetric configuration is unstable and the plate will settle on one of the walls. (**b**) Schematic of a free-to-rotate cylinder with a splitter plate placed in a free stream. The grey region marks the back-flow region. Inside this region the flow (*U*_R_=|**U**_R_|) is constant and in reverse direction to the free stream (*U*=|**U**|). It is postulated that the back flow exerts a similar destabilizing force as the gravity does for the inverted pendulum in **a**. (**c**) The proposed model requires a region of back flow. For a cylinder, we define this region as a half ellipse (see Methods: Back-flow region) with parameters *θ*_0_, and *B*_max_=*B*(0). Also shown is the normal force on the plate inside 
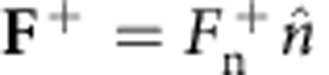
 and outside 
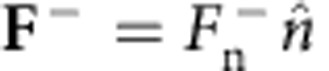
 the back-flow region.

Having established that symmetry breaking is prevalent in the presence of rigid plates for fixed/falling bluff bodies with steady/unsteady wakes (*Re*=45–12,000), we now develop a model that uncovers the underlying instability mechanism. Consider an inverted pendulum system as shown in Fig. 4a that is confined between two walls and free to rotate. Due to the offset between centre-of-mass and fixation point (centre of cylinder), the symmetric straight configuration is unstable and it relaxes to either side of the supporting walls in the presence of any small perturbation. We claim that the physical mechanism for the instability of the symmetric straight configuration of a bluff body with a compact appendage placed in a free stream is similar to the inverted pendulum. The pressure (instead of gravity) in the recirculation zone behind the bluff body acts as the destabilizing force (Fig. 4b).

Consider a steady and uniform free stream *U* and a body consisting of a rigid splitter plate clamped at one point to a circular cylinder (Fig. 4c). The body is free to rotate around the centre of the cylinder, but it may not translate. We assume that the flow is equal to *U* everywhere, except inside a confined back-flow region behind the cylinder. Inside this region the flow, denoted by *U*_R_, is uniform, steady and in opposite direction to *U*. We also assume that the shape of the back-flow region is in the form of an ellipse (Methods: Back-flow region).

### Normal forces on the plate

An inclined plate in a free stream experiences a normal pressure force that depends on the inclination angle *θ*. To find a steady state in our model, part of the splitter plate must be outside the back-flow region and exposed to the free stream *U*, whereas the remaining part is inside the back-flow region and experiences a uniform reversed flow *U*_R_. The length of splitter plate inside the back-flow region is given by a function *B*(*θ*), which depends on the turn angle *θ*. We will show that it is the competition between the forces acting on these two parts of the plate that determines the stability of the system. Since the instability exists under steady conditions (as shown using numerical simulations in Fig. 3b,c), we assume steady forces. Further neglecting viscous forces^18^, the total force on each part of splitter plate due to the fluid can be modelled as


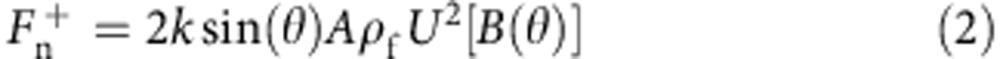


inside the back-flow region and


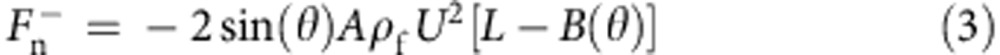


outside the region (Fig. 4c). Here, *A* is a force law calibration coefficient and *ρ*_f_ is the fluid density. The constant coefficient *k*>0 describes the (averaged) magnitude of the force density on the inner part of the splitter plate relative to the outer part. It is shown in the Methods section (Normal force on an inclined plate) that our model of the forces is a special case of a commonly used model for describing the forces on a freely falling plate^19^,^20^.

### The onset of instability

We further assume that the splitter plate is sufficiently thin, such that its weight can be neglected. As a result, the centre-of-mass coincides with the pivot point and the total torque around this point is


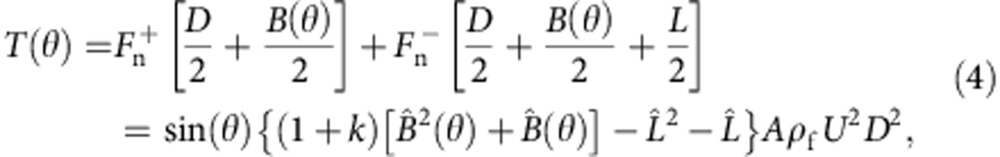


where 
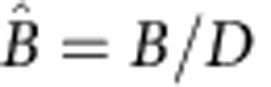
 and 
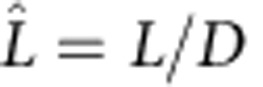
. In our model the condition for the equilibrium state is zero torque. We thus seek turning angles *θ*, for which the total torque on the body vanishes. This condition is satisfied for the trivial straight position *T*(0)=0 and for two non-trivial skewed positions *T*(±*θ*_s_)=0.

The linear stability of the trivial solution is determined by the sign of the first-order term of the Taylor expansion of *T*(*θ*) around *θ*=0, that is,


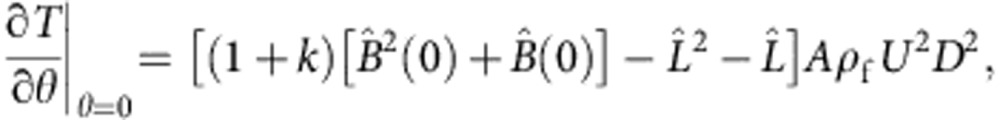


which results in the following condition for instability


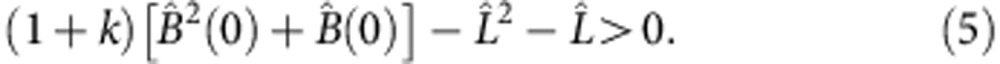


When equation (5) holds, any small deviation from the zero angle *θ*=±*ε*, induces a torque in the direction away from the zero angle. Setting the left-hand side of expression (5) to zero and solving a quadratic equation gives the critical length 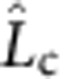 of the splitter plate for the onset of instability,


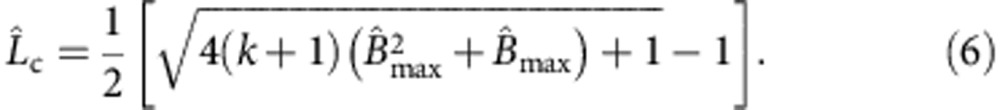


This condition is independent of the coefficient *A*. The two empirical parameters *k* and 
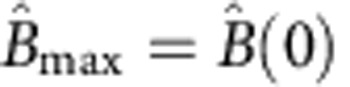
 can be chosen based on wake measurements (see Methods: Model parameters).

At 
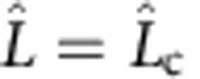
, the straight position *θ*=0 loses its stability, and two attracting states *θ*=±*θ*_s_ appear via a pitchfork bifurcation. These non-trivial equilibria can be found by setting the expression in curly brackets of equation (4) to zero, which results in a quadratic equation for *θ*_*s*_,


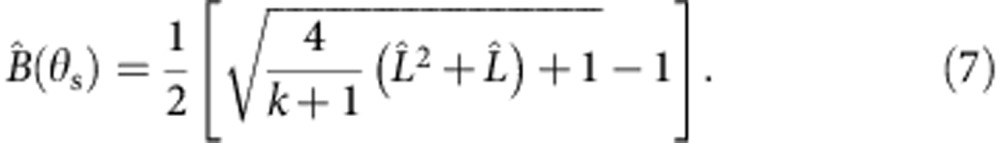


This condition corresponds to a geometrical problem and can be solved analytically for *θ*_*s*_ (Supplementary Note 1 and Supplementary Fig. 9). In Fig. 3b, we compare the predictions of the analytical model with the turn angle of a free-falling body (*Re*=45), where we assert that our model captures the bifurcation with respect to 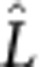 very well. In Fig. 1d, we compare the turn angle based on the steady force law (equations (2) and (3)) with the time-averaged turn angle of the soap-film experiments at *Re*=12,000. We observe a good agreement, which indicates that a steady model is sufficient to capture the instability threshold. In the limit of zero splitter-plate length 
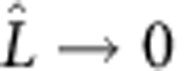
, our model predicts that the stable position is at the angle ±*θ*_0_, for which the flow separates from the body surface (*θ*_0_≈55 degrees for a cylinder). This is in agreement with our experimental (Fig. 1d) and numerical (Fig. 3b) results, where the angle *θ* does not approach zero as 
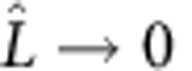
. Our study thus indicates that an arbitrary small protrusion—but larger than the scales of surface irregularities of the cylinder surface—will make the cylinder turn and stabilize at an angle for which the boundary layer on the body detaches from the surface.

### Drift induced by the instability

Our model also predicts the drift angle for a falling body. Due to the asymmetric pressure field at skewed equilibrium turn angles, a non-zero net force, *F*_d_, in the transverse direction to the free stream acts on the object. We may decompose this drift force into a part acting on the cylinder body *F*_cylinder,d_ and a part acting on the splitter plate *F*_plate,d_. The latter contribution can be obtained by projecting forces (equations 2 and 3) on the direction orthogonal to the free stream. The former contribution can be taken into account by assuming *F*_cylinder,d_=*A*_c_*F*_plate,d_, because for any angle *θ*, the cylinder and the plate experience the same pressure field (validation of this assumption based on numerical computations is reported in Supplementary Fig. 2a). Thus, the total drift force on the object can be written as


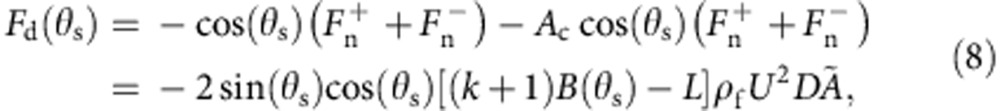


where *Ã*=*A*(1+*A*_c_). This parameter is determined by calibration with numerical simulations (see Methods: Model parameters).

**Figure 5:**
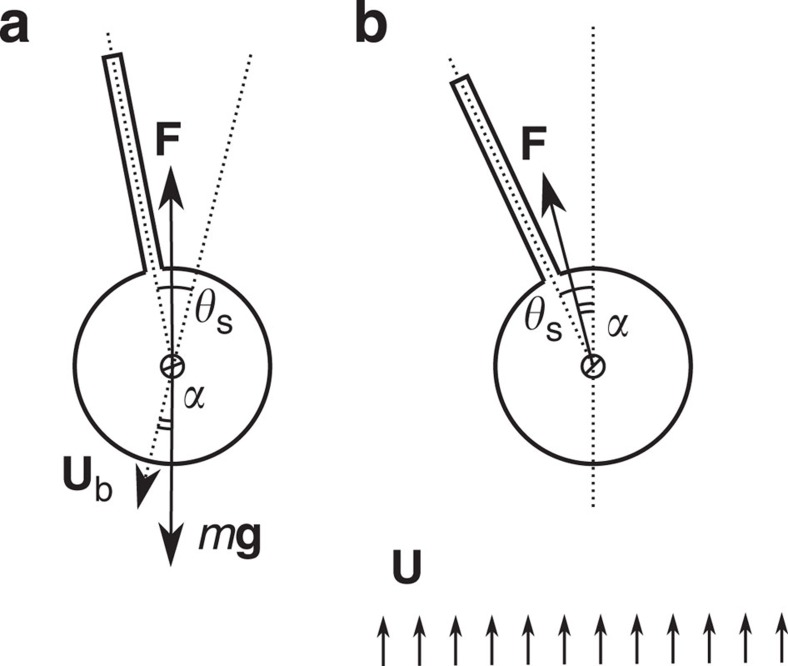
Relation between drift angle and force angle. (**a**) Schematics of a freely falling cylinder with a splitter plate. There is a balance between the force from fluid **F** and the gravitational force *m***g**. The drift and turn angles are denoted by *α* and *θ*_s_, respectively. (**b**) Schematics of a fixed cylinder with a splitter plate subject to a free stream; the force and turn angles are denoted by *α* and *θ*_s_, respectively.

For a freely falling object, the force acting on the body from the fluid must be balanced by the gravitational force (Fig. 5a). The turn angle *θ*_s_ with respect to the direction of movement in the freely falling case is the same as the turn angle *θ*_s_ with respect to the direction of the free stream in the static case (Fig. 5b). The drift angle *α* in the former case thus corresponds to the force angle in the latter case, given by


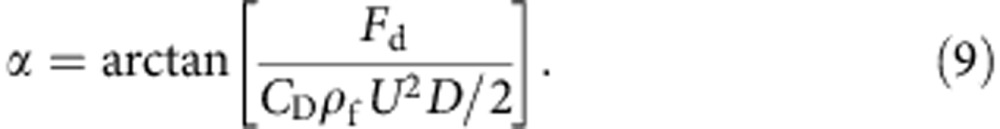


After inserting equation (8) into this expression for *F*_d_ with *Ã*=*C*_D_/4, we obtain


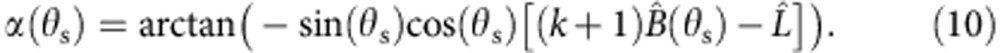


From this expression one finds the drift angle is in the same direction as the splitter plate is tilted (Supplementary Note 2). The direction of drift force can be explained by the fact that the pressure force outside the back-flow region has a larger lever arm than the force inside the region, and thus generates a larger torque *T*_out_. To balance *T*_out_ with the torque on the plate inside the back-flow region, a larger force inside the back-flow region is required to compensate for the smaller lever arm. As such, the force inside the back-flow region is larger than outside and thus determines the drift direction. This is in agreement with our numerical findings; in Fig. 3c we compare the drift angle *α* obtained from (10) with the drift angle from numerical simulations of a freely falling body, where we again observe a good agreement. The drift observed in the soap-film experiments of the hanging body is compared to (10) in Fig. 2d.

So far, we have presented evidence of the IPL instability and its consequence for locomotion in two dimensions and for rigid appendages. Our final results show that the IPL instability is also present and meaningful in three dimensions and for flexible appendages.

### IPL instability for flexible appendages

**Figure 6:**
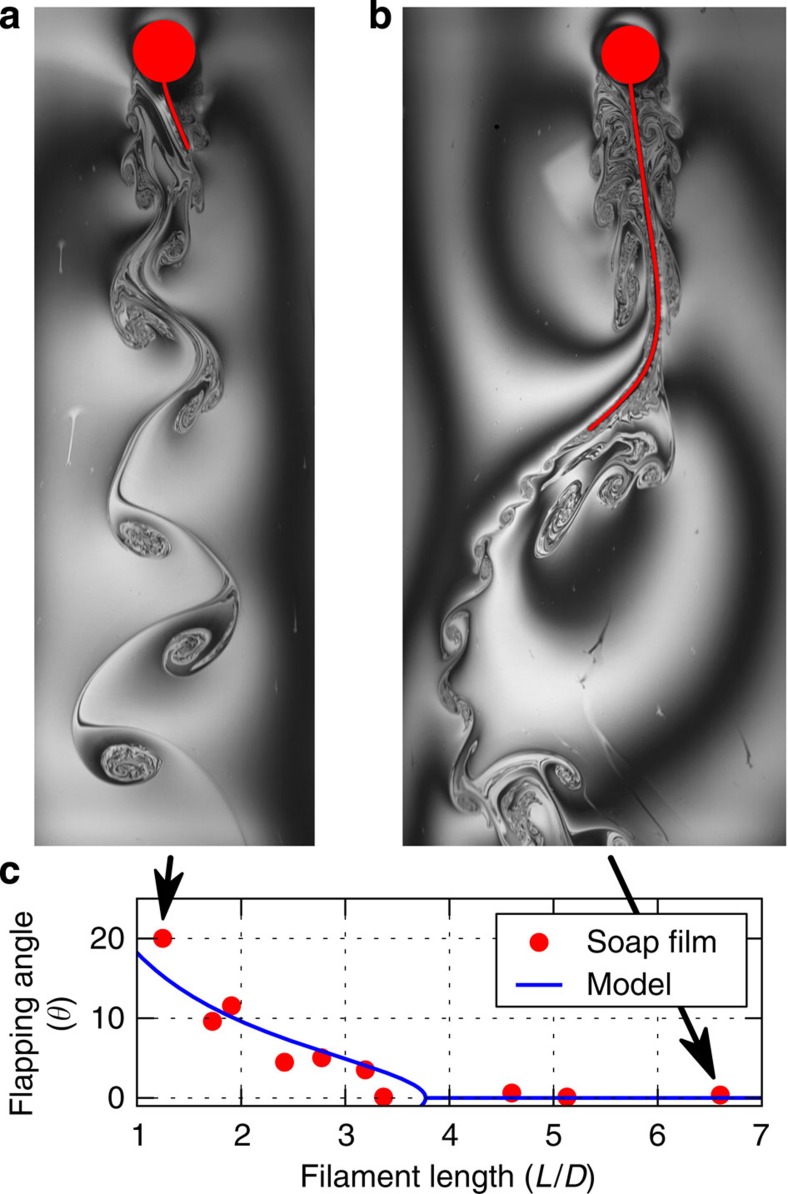
Soap-film experiments of an elastic filament behind a cylinder. Short filament (**a**) oscillates around an asymmetric mean position. In contrast, the long filament (**b**) has a symmetric mean position (Supplementary Movies 4 and 5). Keeping the film velocity fixed at *υ*=2±0.04 m s^−1^ and measuring the average flapping angle for different filament lengths (*L*), a transition from a symmetric flapping to an asymmetric one is observed at *L*_c_=3.3*D*. Blue solid line depicts the predictions of the analytical model (model parameters are the same as in Figs 1d and 2d).

Flexible appendages, often observed on plants and animals, are also susceptible to an IPL instability. We performed soap-film experiments of circular cylinder of diameter *D*=6.88 mm fixed (no rotation or translation allowed) at its centre with a flexible filament (silk) attached to its rear end (see Methods: Soap-film experiments of a fixed body). When the filament is sufficiently long, the inertial and elastic forces of the filament interact with the fluid pressure, causing the filament to flap (Fig. 6b). This archetype of fluid–structure interaction problem is often used as a model of flag fluttering^6^,^21^,^22^. The time-average mean position of the long filament is a vertical straight position, and therefore the flapping is symmetric. However, due to the IPL instability, filaments shorter than a critical length (*L*_c_=3.3*D*) have an asymmetric mean position (Fig. 6a) that is sustained for all times. This is manifested by a non-zero average angle *θ* formed by the vertical centre axis with the straight line connecting the filament anchor point to its tail position. The elastic appendage undergoes the same type of bifurcation (Fig. 6c) as observed for the splitter-plate configuration, and is surprisingly well captured by our rigid-plate model.

### IPL instability for three-dimensional bodies

**Figure 7:**
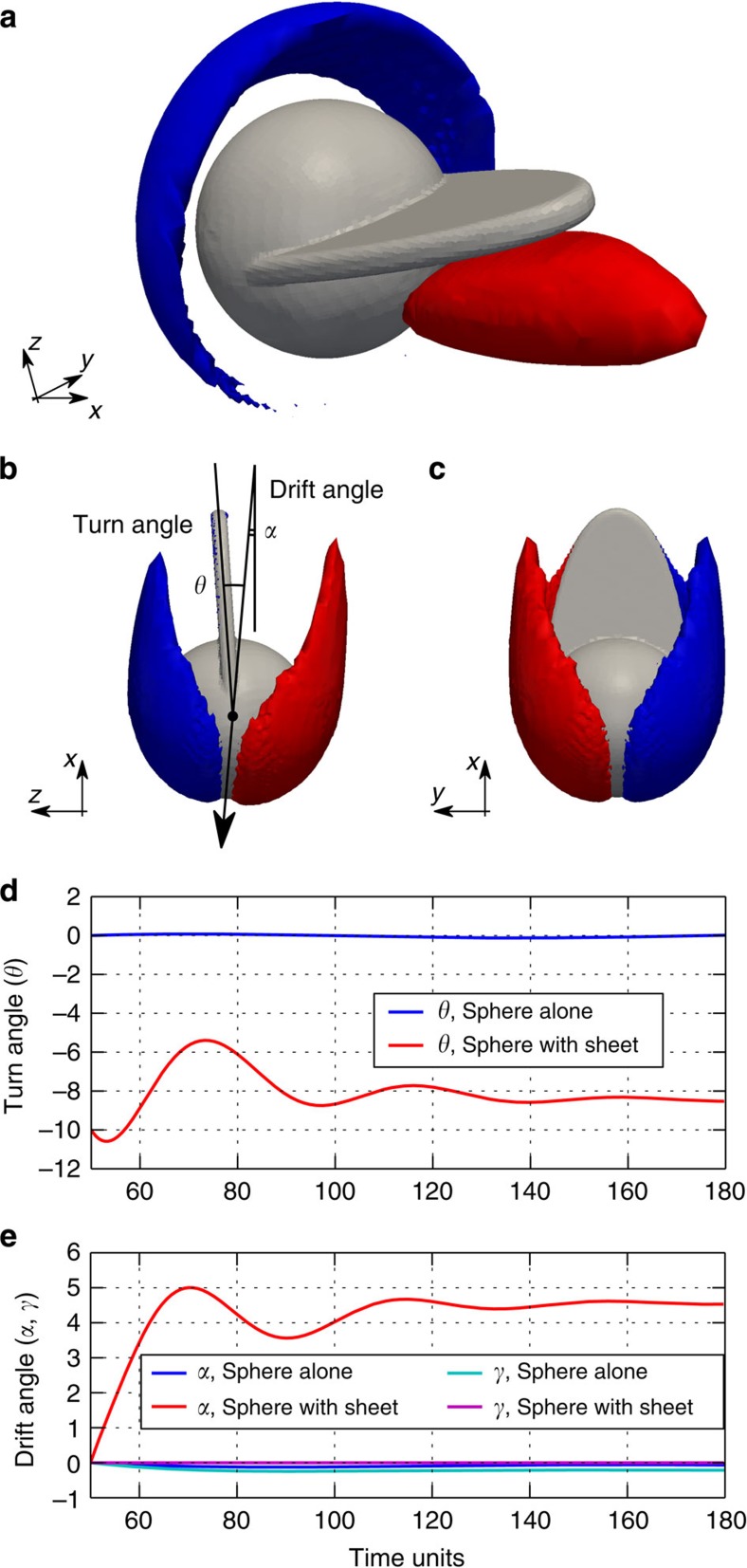
Numerical simulations of a sphere with a sheet. We model a freely falling object (shown in grey colour in frames **a**–**c**) by imposing a free stream in the *x*-direction and allowing translation in *y* and *z* directions, as well as rotation around *y* axis. In **a** the *u*=−0.1*U* streamwise-velocity isosurface (red) shows the existence of a back-flow region, whereas *u*=1.1*U* isosurface (blue) illustrates the asymmetric flow field around the sphere. The asymmetric wake is also observed in **b**, where the vorticity component in the *y*-direction is shown (blue and red correspond to *ω*_*y*_=3.0 and *ω*_*y*_=−3.0, respectively). The direction of movement of the body is depicted with a black arrow. In contrast, in the *xy*-plane shown in **c** there is no drift and the wake is symmetric (blue and red corresponding to *ω*_*z*_=3.0 and *ω*_*z*_=−3.0, respectively). The time evolution of the turn angle (*θ*) and the drift angles (*α*,*γ*) for the sphere alone and the sphere with a sheet are reported in **d** and **e**. When a sheet is appended to the body it rotates an angle of *θ*=−8.5 degrees and drifts in *zx*-plane by *α*=4.5 degrees. See also Supplementary Movies 6 and 7.

Using direct numerical simulations, we show that a three-dimensional object undergoes an IPL instability with an ensuing side force on the body. The object under consideration has a density ratio *ρ*_s_/*ρ*_f_ =5 and is composed of a sphere of diameter *D* and a thin elliptic-shaped sheet (Fig. 7a–c). The sheet is attached to the sphere and protrudes a maximum length of *L*=0.8*D* from the rear stagnation point of the sphere (Supplementary Fig. 3). The object is subject to a constant free stream *U* in the streamwise direction *x*. We allow the body to rotate around the transverse axis *y* and to translate in the *yz*-plane. The three degrees of freedom of the body can be described by the angles *θ*, *α* and *γ*. The two former angles correspond to the turn angle and to the drift angle in the *zx*-plane (Fig. 7b), that is, *θ* is the deviation of the sheet from the direction of movement in the *zx*-plane and *α* is the angle formed between the velocity of the object in the *z*-direction and the free-stream velocity *U* in the *x*-direction. Similarly, the angle formed between the velocity in the *y*-direction and *U* is denoted by *γ*.

At *Re*=*UD*/*ν*=200 a steady axisymmetric wake^23^ forms behind the sphere alone (no sheet attached). Due to symmetry, the sphere neither rotates nor drifts (Fig. 7d,e). According to the IPL model and our two-dimensional investigations, we expect that by adding an appropriate protrusion to the sphere, the object will rotate and experience a non-zero transverse force. Indeed, in the presence of the elliptic sheet, we observe that after a transient time, the sphere stabilizes at a turn angle of *θ*=−8.5 degrees (Fig. 7d) and drifts with a constant velocity in the *zx*-plane with an angle of *α*=4.5 degrees (Fig. 7e). We observe a zero drift in the *xy*-plane, that is, *γ*=0.0 degrees (Fig. 7e). The drift is a consequence of the IPL instability: any small perturbation causes the sheet to move away from the straight unstable position (*θ*=0) and to settle on a skewed stable angle *θ*_s_. The new equilibrium breaks the symmetry of the wake (Fig. 7b) in the *zx*-plane, which in turn induces a side force on the body in the *z*-direction, making it drift. Note that the trait of the IPL-induced movement—the direction of drift and the direction that the appendage is titled in are the same—is present. Although the chosen three-dimensional appendage triggers the IPL instability, its size and shape have not been optimized to yield maximum drift.

## Discussion

There exist many motile animals with splitter-plate-shaped appendages^2^,^10^,^11^,^12^,^13^. While the presence of a splitter plate has for a long time been associated to a ‘trick’ to reduce drag on bodies^2^, it has not until the present work been associated to the generation of rotation and drift. We can postulate two requirements that need to be fulfilled for an organism to make use of the IPL instability for locomotion. First, when the organism moves in the fluid, a separated region has to be formed around its body. This requirement excludes very small organisms, where fluid inertia is negligible, that is, the Stokes flow regime. Second, the passive appendage needs to be sufficiently short, such that a significant portion of its area is exposed to a reversed flow. We have shown that the instability is a two-dimensional mechanism, but that the induced drift is equally significant in three dimensions as in two. We expect that the consequences for locomotion can be even more significant if the three-dimensional shape of an appendage is optimized to yield maximum drift. In particular, it would be interesting to investigate whether these optimized shapes resemble appendages that have evolved naturally.

In conclusion, we identify a new mechanism for locomotion; within a biologically well-inhabited domain of parameters (*Re*=45–12,000, covering steady and unsteady wakes) moving two- or three-dimensional bodies with short rigid or flexible protrusions are likely to undergo an IPL instability. We believe that these results form a foundation, from which scientists can discover the existence of the IPL instability in various forms in nature. The beauty of this passive locomotion technique is that no energy needs to be expended by the animal; instead the existing energy in the flow is used.

## Methods

### Soap-film experiments of a fixed body

The experiment is performed with a gravity-driven soap film located at KTH in Stockholm (Fig. 1a). The typical size of the test section is 1.2 m long and 8 cm wide. The fluid velocity is varied between 1 and 3 m s^−1^, and the corresponding film thickness varies from 1 to 4 μm. The fluid velocity—measured with Laser Doppler velocimetry—is close to uniform at the centre of the channel (the variation of the fluid velocity was below ±2.5% over 70% of the channel span). The diameter of the cylinder is around 6.5 mm and the length of the filament/splitter plate varies from 6 to 50 mm. For the cylinder–flexible filament system, a fixed cylinder made of plexiglass puncture the film; the filament—a silk fibre with a diameter of 0.25 mm—pass through a hole at the back of the cylinder. The bending stiffness of the silk filament is determined by measuring its Young’s modulus with a tensile test. Using the area moment of inertia of the filament, the bending stiffness is found to be 0.04 erg cm^−1^. The cylinder–splitter plate are made with a plastic sheet (0.1 mm thick); the cylinder is free to rotate around a solid axis passing through its centre. For the visualization of the flow a low-pressure sodium lamp is used, and the resulting interference fringes are filmed with a high-speed camera at 500 Hz, the resolution of the camera is 2,048 × 2,048 pixels, 1,500 images are taken for each test corresponding to a recorded time of 1.5 s. To determine the position of the end of the filament/splitter plate the recorded grey-scale images are binarized in black and white images using a threshold in which the filament/splitter plate is black and the background is white. The position of the end of the filament/splitter plate are then averaged in time to determine the mean position and turn angle of the filament/splitter plate.

### Soap-film experiments of a hanging body

The experiment is performed with a gravity-driven soap film in Bordeaux. The setup in terms of the typical size of the test section, the fluid velocity and film are the same as in the soap-film experiments of the fixed body, except that for the visualization of the flow a white lamp is used. We use cylinder with diameter *D*=7 mm, to which splitter length with length *L*=2.1*D* is attached. We fixed it on a ‘loose’ pendulum (see Fig. 2a) made of a thin nylon wire. This wire crosses the film perpendicularly through a small hole drilled in the centre of the disk. The system, consisting of a disk and splitter plate, is free to rotate under such conditions. We estimate the drift force *F*_d_ from a force-balance equation. When the pendulum has reached equilibrium, the following forces act on the cylinder and splitter-plate system (see Supplementary Fig. 4b): the drift force **F**_d_, the drag on the system **F**_drag_ (we approximate it with the drag of cylinder alone, see Supplementary Fig. 1), the weight of the disk **P** and the tension of the wire **T**. At equilibrium, the torque around the fixation point of the pendulum is zero. Neglecting the wire weight (which means underestimating the drift force), this condition leads to,


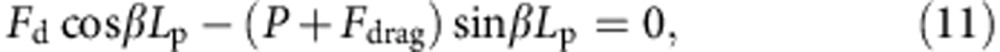


where *L*_p_ is the length of the pendulum and *β* is the deviation angle of the pendulum. We may calculate *F*_d_ as


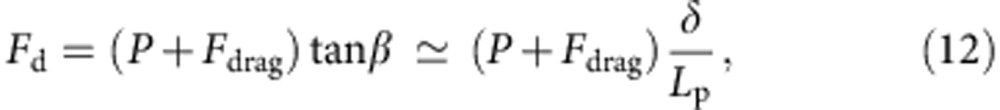


where *δ* is the displacement (see Supplementary Fig. 4b) between the position given by the symmetric state (Fig. 2b) and the position of the disk in the asymmetric state (Fig. 2c). Thus, to determine *F*_d_, we measured *δ* and *P* and estimated *F*_drag_. Using those measurements, we obtain the force angle, shown in Fig. 2d.

### Numerical simulations of a two-dimensional body

We discretize the two-dimensional incompressible Navier–Stokes equations with a staggered-grid, finite-volume formulation using a second-order semi-implicit time integration scheme. The no-slip boundary condition is enforced at Lagrangian points by appropriate regularized surface forces^24^. A uniform grid size of *h*=1/25 dimensionless length units is sufficient to reproduce previous work^25^ on freely falling circular cylinder in terms of trajectory. The dimensionless length is the diameter of the cylinder, *D*. The equations for the motion of the rigid body are coupled to fluid solver implicitly. This ensures numerical stability at density ratios between solid and fluid as low as *ρ*_s_/*ρ*_f_=1.0001. The resulting linear equation system is solved using approximation of block-LU decomposition^24^ and direct solver^26^. The freely falling cylinder with the splitter plate is placed in a large computational box with no-slip boundary conditions imposed at the walls. The box dimensions depend on the duration of transient behaviour; typical size used is 40*D* in width and 110*D* in height. A uniform cartesian grid covers a large region, typical size is 20*D* wide and 90*D* high. In the region between the uniform mesh and domain boundary, the mesh is expanded smoothly. The no-slip boundary condition at the box wall is always at least 10*D* away from the body. The time-dependent simulation is continued until it reaches a terminal motion. The angle of drift is obtained using linear regression of the trajectory in the terminal regime. The obtained angle is compared with corresponding simulation of the cylinder alone, using the same mesh and the same initial conditions. The cylinder alone has a small drift (around 0.3 degrees) due to boundary effects. To increase the accuracy, we subtract the unphysical drift from the results of cylinder with splitter plate for all lengths. The angle of splitter-plate orientation is determined as a mean over terminal regime.

### Back-flow region

In the proposed model, the back-flow region defines the boundary between two sections of the plate; the two sections experience a total normal force in opposite directions. Thus, the underlying assumption of our model is that the normal force on the plate from the fluid changes sign at some distance from the cylinder surface. Indeed, our two-dimensional numerical computations verify that when a plate of a given length stabilizes at a particular angle, there exists a point on the plate for which the normal force changes sign. In Supplementary Fig. 5a, we show with black stars these points for different plate lengths. Ideally, these points would define the function *B*(*θ*), however, one does not always have a detailed information about force distribution over an appendage available. Therefore, in what follows we suggest a way to approximate the function *B*(*θ*) with measurements of the wake in the absence of a plate.

In Supplementary Fig. 5b streamlines (black lines) of the flow past a two-dimensional circular cylinder at *Re*=45 are shown. The unperturbed wake consists of two steady symmetrical vortices and the length of the recirculation bubble *L*_w_ is around 2.5*D* (measured from the rear stagnation point). In the same figure, we show contours (green lines) of zero azimuthal velocity *u*_*θ*_, defined as


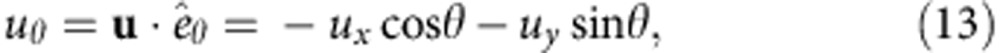


where *ê*_*θ*_=(−cos*θ*, sin*θ*) is the azimuthal unit vector (as defined in Supplementary Fig. 5d), and **u**=(*u*_*x*_,*u*_*y*_) is the velocity field. Excluding the vertical centre line, we observe that *u*_*θ*_ =0 encloses a region with a length about 1.2*D*. This region is compared with the one obtained from the numerical force distribution (black stars) in Supplementary Fig. 5c, where we observe that the two regions are similar in shape. We have approximated this shape with a half ellipse.

### Normal force on an inclined plate

Our starting point for modelling the forces on the splitter plate attached to the cylinder surface is the same as the force model for freely falling rigid plates^19^,^20^. To simplify the problem of a moving plate in a still fluid, we consider the plate in a translating coordinate system as shown in Supplementary Fig. 6a. This transformation results in a fixed plate subject to an incoming free stream. The direction of the free stream is from bottom to top (in *ŷ* direction) to better resemble a free-falling motion. Then, following the work of Andersen *et al.*^27^, we write the drag force **F**_D_ and lift force **F**_L_ on the plate as


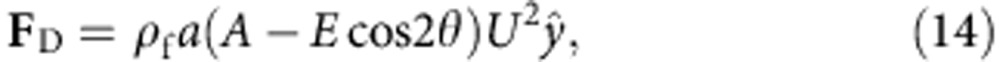



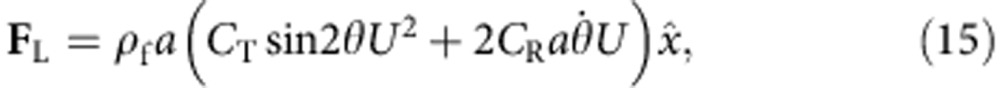


where *ρ*_f_ is the density of the fluid, *a* is the length of the plate, *A*, *E*, *C*_T_ and *C*_R_ are constants, 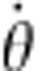 is the angular velocity and *U*=|**U**| is the flow speed. The drag force is an empirical formulation proportional to velocity square and the turn angle, as suggested by Wang *et al.*^28^ The lift force is based on the empirical model for circulation


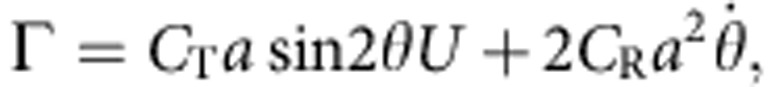


developed by Pesavento and Wang^29^. The above model of forces may be used to describe the motion of the plate (with additional models of added mass and the plate inertia). The coefficients *A*, *E*, *C*_T_ and *C*_R_ are usually calibrated to fit experimental or numerical data of the trajectory of the falling body.

The normal force component can be obtained by projection of lift and drag forces


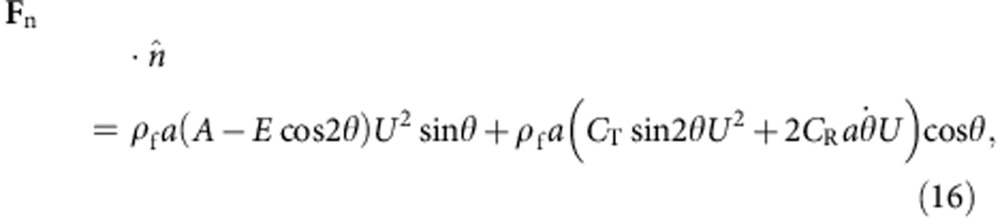


where the normal unit vector 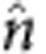 is defined in Supplementary Fig. 6. We simplify this expression by assuming steady conditions and zero contribution from viscous forces (see Supplementary Fig. 7 and associated discussion below). In a steady configuration, 
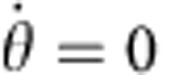
, which renders the second term on the right-hand side in the lift force (15) zero. In the absence of viscous forces, the tangential force component **F**_*τ*_ is zero. When *θ*=0,


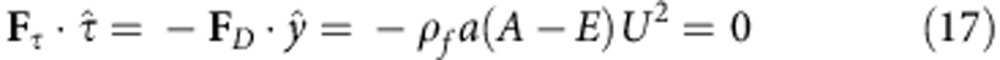


which results in the condition *E*=*A*. When *θ*≠0, we have


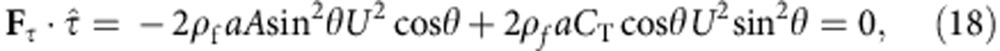


where the trigonometric identities sin2*θ*=2sin*θ* cos*θ* and 1−cos2*θ*=2sin^2^*θ* have been used. This expression is zero for *C*_T_=*A*.

Inserting the conditions 
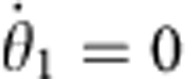
, *C*_*T*_=*A* and *E*=*A* in (16), the normal force component (which now is also the total force on the plate) can be written as


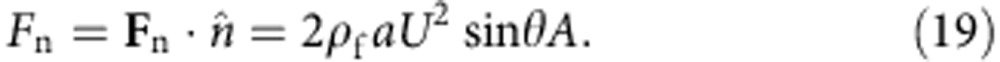


To model the forces on a plate attached to the rear end of a cylinder, we assume that a section of the plate (near the cylinder surface) with length *a*_1_=*B*(*θ*) experiences a reversed flow 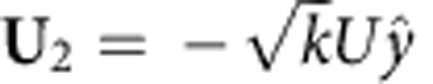; the outer section of the plate of length *a*_2_=*L*−*B*(*θ*) is exposed to the incoming free stream **U**_1_=*Uŷ*. As shown in Supplementary Fig. 6b, the inclined plate experiences uniform steady flows from two directions. Then, by considering the two sections separately, we arrive with the model of normal forces in equations (2) and (3). In this model, *k* defines a constant scaling between the force on the outer side and the inner side of the splitter plate.

Let us assess our force model (equations (2) and (3)) by comparing the torque *T*(*θ*) around the body from our model (equation (4)) with the torque extracted from numerical simulations of a body fixed at various turn angles (*Re*=45). Supplementary Figure 7 shows that by calibrating *A* appropriately (in this case *A*=*C*_D_/16=0.094), our force law provides a reasonable model of the numerically computed torque. Moreover, in Supplementary Fig. 7, we report on the magnitude of the viscous torque in the generated total torque. We observe that the contribution of the viscous component is smaller than the pressure component; using pressure force alone to compute torque introduces less than one degree error in the equilibrium turn angle. This observation underlies our assumption of zero contribution of viscous forces to the total force. Note, however, that viscosity is necessary to induce boundary layer separation. Thus, while we neglect the viscous component in the total force acting on the plate, our model takes viscosity into account implicitly by modelling a back-flow region.

### Model parameters

Here, we propose to determine parameters *B*_max_, *k* and *θ*_0_ from measurements of the wake behind the body without an appendage and to calibrate the coefficient *Ã* with measurement of the drift force on the body. Let us start with *B*_max_ by considering the azimuthal velocity of the unperturbed wake, which is zero along the centre line. For a two-dimensional flow the derivative of the azimuthal velocity on centre line is given by


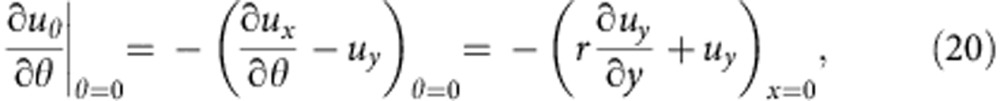


where the continuity equation (∂*u*_*x*_/∂*x*=−∂*u*_*y*_/∂*y*) and coordinate relation (∂/∂*θ*=−*r*∂/∂*x* at *θ*=0) have been used. In Supplementary Fig. 8a,b we see that the derivative of the azimuthal velocity changes sign along the centre line. One can conclude that the point on the centre line, where the derivative of the azimuthal velocity changes sign, corresponds to the point where the centre line intersects with the (off-centre) zero isoline of azimuthal velocity. This is the point that we suggest to use for determining the value of 
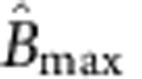
. In Supplementary Fig. 8a, we show ∂_*θ*_*u*_*θ*_ for the flow past a cylinder at *Re*=45, where one observes that 
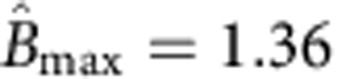
. In Fig. 3b, using the slightly smaller value of 
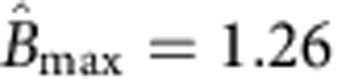
 provided the same critical value *L*_*c*_ as the full numerical simulations. Thus by combining measurements of the cylinder wake without a protrusion and our model, one may predict the critical value for bifurcation with a 90% accuracy. Regarding the soap-film experiments, we measure the vertical velocity component along the centre line, and use its time-averaged value to obtain ∂_*θ*_*u*_*θ*_ from equation (20). The derivative of the azimuthal velocity obtained from experiments is shown in Supplementary Fig. 8b, where a change of sign is observed at 
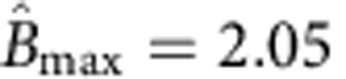
; for model predictions in Fig. 2d, Fig. 1d and Fig. 6c, the length was 
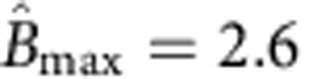
. Thus, the accuracy of wake measurements is around 70%.

We propose to obtain the value *k* by estimating the forcing inside and outside of the back-flow region. Using the definitions of 
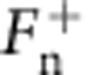
 and 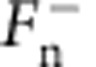 and choosing *L*=*L*_w_ as a representative splitter-plate length, we may estimate *k* from


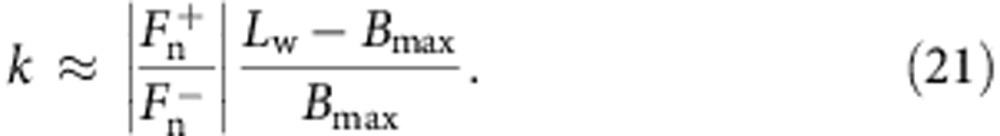


We aim to estimate *k* from a wake without an appendage. It is not possible to measure these forces directly, since they do not exist before the introduction of the splitter plate; therefore we suggest to use measurements of the azimuthal velocity close to the straight position (*θ*=0) to estimate the force ratio. Since *u*_*θ*_ approaches zero as *θ*→0, we assume direct proportionality


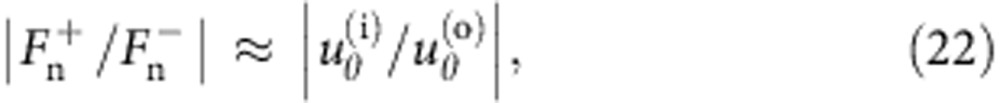


where


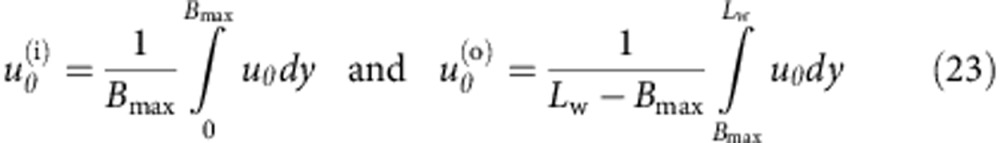


are the average azimuthal velocities inside and outside of the BFR. The limit can be found as


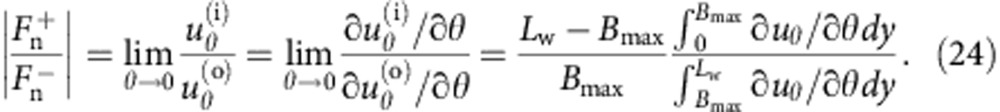


Evaluating the above integrals for the numerical profile (Supplementary Fig. 8a), we obtain *k*=0.83. We used a slightly larger value of *k*=0.9 in our model. The discrepancy is small when taking into account the number of assumptions employed to derive the model. The integral of the experimental profile (Supplementary Fig. 8b) results in *k*=0.18, whereas the value *k*=1.0 was used in the model predictions in Fig. 2d, Fig. 1d and Fig. 6c. We are thus able to determine the order of magnitude of the forcing coefficient *k* from local measurements of one velocity component.

Moreover, from measurements we observe that the angle for which the boundary layer separates from the cylinder is close to *θ*_0_≈55 degrees for the range of Reynolds numbers under investigation.

Finally, to determine the coefficient *Ã*, we calibrate our model with the drift force *F*_d_ extracted from numerical simulations at *Re*=45 of the whole body (cylinder and splitter plate) fixed at various turn angles. The numerical force is compared with the drift force (equation (8)) predicted from our model in Supplementary Fig. 2b for *Ã*=*C*_D_/4, where *C*_D_=1.5 is the drag coefficient of the whole body.

### Numerical simulations of a three-dimensional body

We use the open-source software OpenFOAM^30^ to solve the three-dimensional incompressible Navier–Stokes equations. A sphere is placed in a computational box, which is *L*_*x*_=40*D* long, *L*_*y*_=30*D* wide and *L*_*z*_=40*D* high. Here *D* denotes the sphere diameter, which also serves as a reference unit length. The coordinates for the sphere centre are (15,15,20)*D*. A uniform velocity is imposed at the inflow and a Neumann condition at the outflow, whereas on the lateral sides of the box a slip boundary condition is enforced. No-slip boundary condition are imposed on the object. A hexahedral-dominant mesh is generated using the utilities blockMesh and snappyHexMesh. The former generates a uniform cartesian mesh, while the latter inserts the geometry of the body and refines the mesh locally. The final mesh consists of around 750,000 cells. The chosen solver is pimpleFoam and its extension for dynamic meshes pimpleDyMFoam. This solver makes use of a blend of PIMPLE and PISO algorithms to handle the pressure–velocity coupling, together with an adaptive choice of the timestep under a maximum Courant number condition. To compute the rigid motion of the body, we use the sixDoFRigidBodyMotion solver, which also handles the dynamic mesh (by rotating and stretching the cells according to the body motion^31^). The coupling between the fluid and the body is solved with an explicit scheme, that is, using the so-called weak-coupling approach. To validate the solver, we performed a simulation of the fixed sphere at *Re*=200 and obtained a good agreement in terms of the wake length and drag coefficients with previous studies^23^. We complement the sphere with an elliptic sheet (length behind sphere is 0.8*D*, thickness of sheet is 0.1*D*). The design of the appendage is shown in the Supplementary Fig. 3. We use the same mesh settings and the same computational box, which was verified using the simulation of the sphere alone. The length of the simulation is limited due to large displacement of mesh towards the end of simulation, when the body approaches the boundaries of the box. To partially overcome this limitation, we fix the body for the first 50 time units and then allow it to move. In this way, most of the transient dynamics of the flow can be simulated when the object is at the initial position and no mesh deformation is required.

## Author contributions

S.B. and A.M. conceived the original idea. S.B. initiated and supervised the research. N.B. performed the soap-film experiments of the fixed cylinder with feedback from F.L. F.I. and H.K. performed the soap-film experiments of the free-hanging cylinder. U.L. performed the numerical simulations of the two-dimensional free-falling cylinder. A.M. supervised the numerical simulations of the three-dimensional sphere. U.L. created the theoretical model, which he further developed with feedback from all authors. All authors analysed data. S.B. and U.L. wrote the paper.

## Additional information

**How to cite this article:** Lācis, U. *et al.* Passive appendages generate drift through symmetry breaking. *Nat. Commun.* 5:5310 doi: 10.1038/ncomms6310 (2014).

## Supplementary Material

Supplementary Information Supplementary Figures 1-9 and Supplementary Notes 1-2

Supplementary Movie 1 This movie shows soap-film experiments of a disc and splitter plate suspended to a pendulum as the film velocity is gradually increased. Initially, for low velocities, the body is in a symmetric state, but as the velocity is increased, the system becomes unstable. This results in a turn of the body as well as - because the disk splitter hang freely - a drift to the right (in the same direction as the tilt of the splitter).

Supplementary Movie 2 The experimental setup is the same as in Supplementary Movie 1, but at a fixed film velocity for which the disk plus splitter is always in an asymmetric state. Initially, the splitter points to right. A needle is then brought close to the splitter plate and causes a perturbation of the flow. The splitter plate then changes angle and points to the left. The disk slowly drifts to the left as expected.

Supplementary Movie 3 In this movie we show a contour plot of the vorticity field (levels from -3.0 to 3.0) obtained from numerical simulations of a freely falling body (main paper Fig.3a) at Re = 156, L= 1.0 and ρ= 1.01. To demonstrate the drift of the body, the initial position is marked with red dot and the trajectory with a red line.

Supplementary Movie 4 We show soap-film experiments (film velocity v = 2 m/s) of a silk thread of length L = 6.6D attached to the rear of a cylinder of diameter D = 6.88 mm. The time average position of the flapping filament is a straight vertical line, rendering its motion symmetric.

Supplementary Movie 5 Same experiment as in Supplementary Movie 4, but now with a silk thread of length L = 1.26D, which is below the critical value for instability Lc = 3.3D. The motion of the filament is constraint to the right side of the recirculation zone.

Supplementary Movie 6 The animation shows vorticity contours of a three-dimensional sphere in the zx-plane. To model a free-falling object, a free-stream is imposed in the xdirection, and the object is allowed to translate in y and z directions as well as to rotate around the y-axis. The three degree-of-freedom rigid-body motion is enabled at t = 50, where it is observed that the body experience a very small drift to right (order 0.1 degrees) due to transient effects as well as asymmetries arising due to numerical approximations (in particular the computational grid).

Supplementary Movie 7 Same numerical experiment as in Supplementary Movie 6, but now with an elliptic-shaped sheet of length L = 0.8D attached to the sphere. A clear drift to the left (order 1 degree) is observed. The appendage points to the left, as expected when bodies experience an IPL instability.
