# Supplementary material for: Passive appendages generate drift through symmetry breaking
Source: Nat Commun. 2014 Oct 30;5:5310. doi: 10.1038/ncomms6310 (PMC4220513; doi:10.1038/ncomms6310)
Supplement: Supplementary Information — Supplementary Figures 1-9 and Supplementary Notes 1-2 [file ncomms6310-s1.pdf]

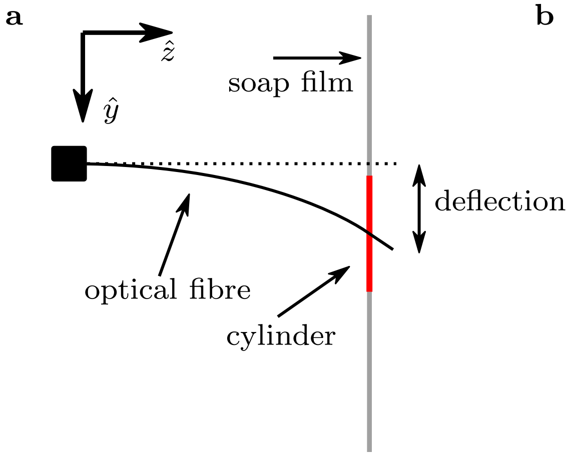

Supplementary Figure 1: Measurement of the drag force. Sketch of the cylinder in the soap film, fixed to a calibrated cantilever made of a thin optical fibre. A side view (**a**) and a front view (**b**) is shown. The forces acting on the cylinder are indicated in the front view (**b**).

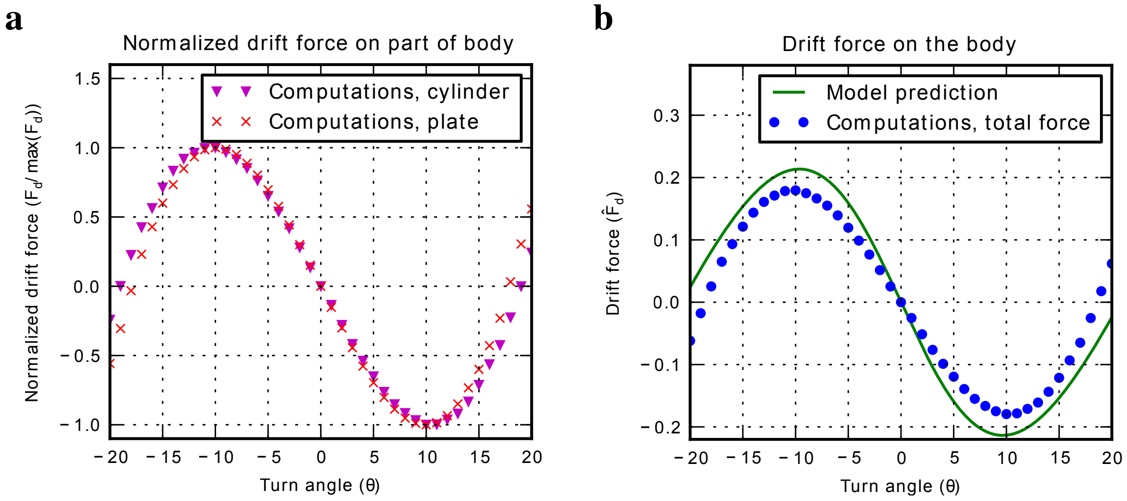

Supplementary Figure 2: Drift force on a fixed body at various turn angles. The drift force $\hat{F}_{\rm d}(\theta) = F_{\rm d}(\theta)/(1/2\rho_{\rm f}U^2D)$ is extracted from numerical simulations of a flow ($Re = 45$) around a cylinder with splitter plate of length $L = 1.0D$ at turn angles $-20^\circ < \theta < 20^\circ$. In **a** we compare the angular dependence of the drift force for the plate with the drift force of the cylinder. The forces are normalized with their maximum values in order to show that they have a similar shape. In **b**, the drift force is compared to the force obtained from the model for $\tilde{A} = C_D/4$.

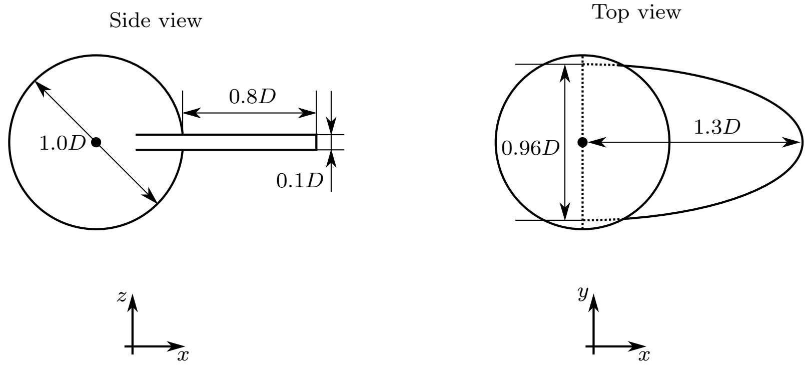

Supplementary Figure 3: Sketch of the three-dimensional object. A side view is shown in the left frame and a top view is shown in the right frame. The sheet behind the sphere is designed as half of an ellipse with minor axis aligned with the diameter of the sphere. The thickness is chosen to be relatively small in order not to shift the center of mass too far from the center of the cylinder. Sharp corners are smoothened using curvature with radius $r = 0.04D$.

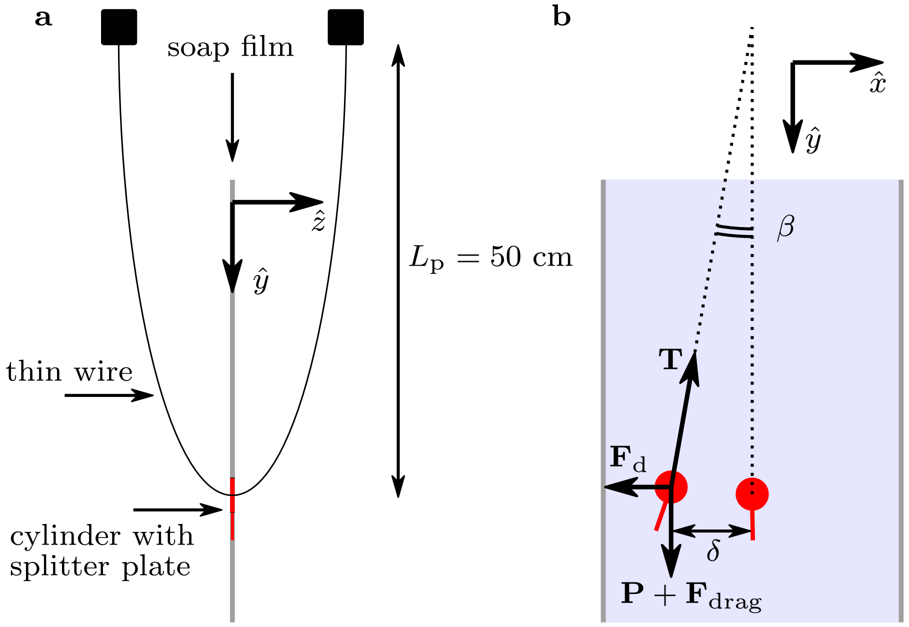

Supplementary Figure 4: Sketches of the splitter plate, fixed to a pendulum. In **a** the side view is shown and in **b** the front view is shown. The different forces acting on the system are indicated on the front view (**b**).

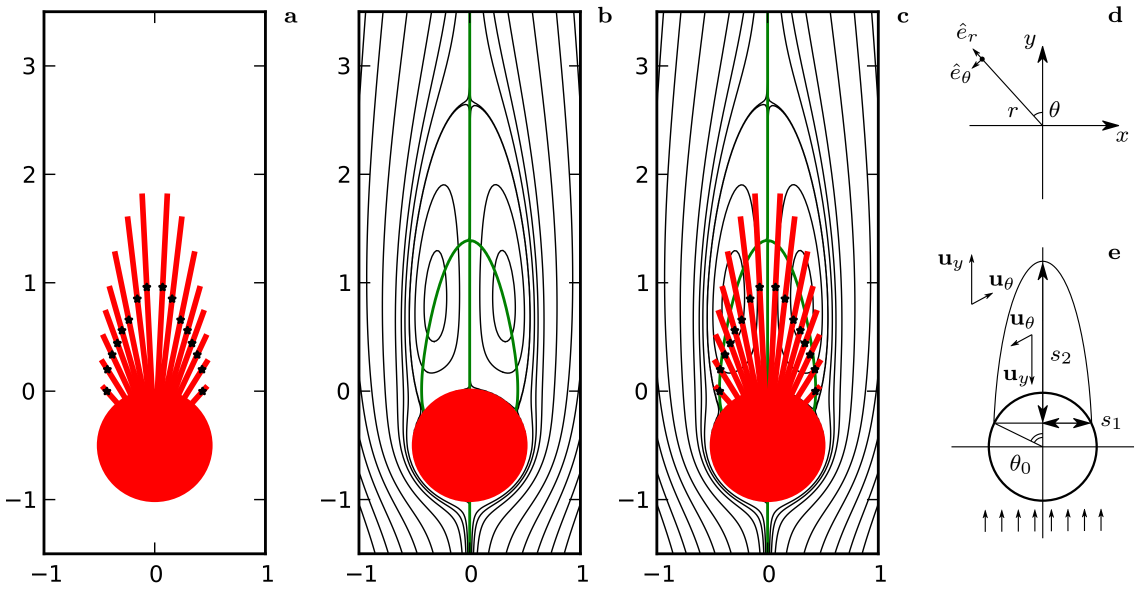

Supplementary Figure 5: Definition of model back flow region for cylinder. a, Black stars mark the position where the normal force on splitter plates of different lengths changes direction. **b**, Flow field around cylinder at Re = 45 is shown. Black lines depict streamlines and green lines contours of zero azimuthal velocity. **c,** Superposition of splitter plates at selected equilibrium angles (**a**) and the undisturbed flow around cylinder (**b**) are shown. **d,** The definition of cylindrical coordinate system. Note that zero angle direction is aligned with the $\hat{y}$ direction. **e,** The half ellipse of back flow region is defined using major semi-axis s_2 and attachment angle θ_0, which sets the minor semi-axis s_1. We only consider shapes with different length of s_2, keeping θ_0 constant. In **e,** we also show the direction of azimuthal velocity $\mathbf{u}_\theta$ on the left side of the domain. On the right side of the domain, u_θ has the opposite direction.

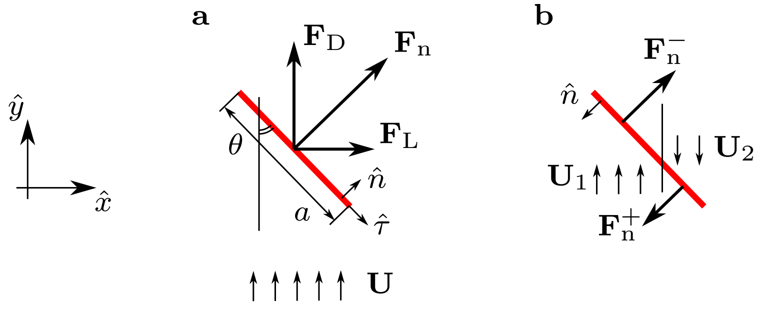

Supplementary Figure 6: Normal force on the plate. a, The problem of a moving plate can be described in a coordinate system where the plate is not translating but only rotating. In this frame of reference, the plate is exposed to incoming free stream with velocity $\mathbf{U}$. The angle between the plate and the incoming flow is θ. The angle θ and flow velocity $\mathbf{U}$ can be time dependent. **b,** The simplified force model applied on a plate, which is divided in two parts; one part is exposed to the free stream $\mathbf{U}_1$; and the other part is exposed to free stream $\mathbf{U}_2$ in the opposite direction.

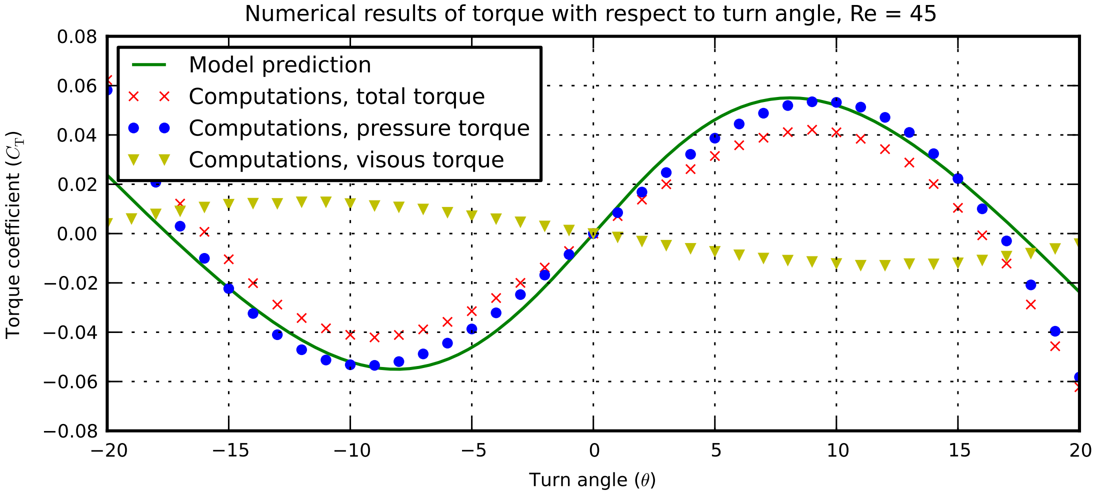

Supplementary Figure 7: Torque on a fixed body at various turn angles. The torque coefficient $C_{\mathrm{T}} = T/(1/2\rho_{\mathrm{f}}U^2D^2)$ is extracted from numerical simulations ($Re = 45$) of a flow around a cylinder with splitter plate of length $L = 1.0D$ at turn angles $-20° < \theta < 20°$. The torque coefficient is compared to the torque obtained from the model when $A = 0.094$. The viscous contribution of the coefficient is also shown (with triangle symbols).

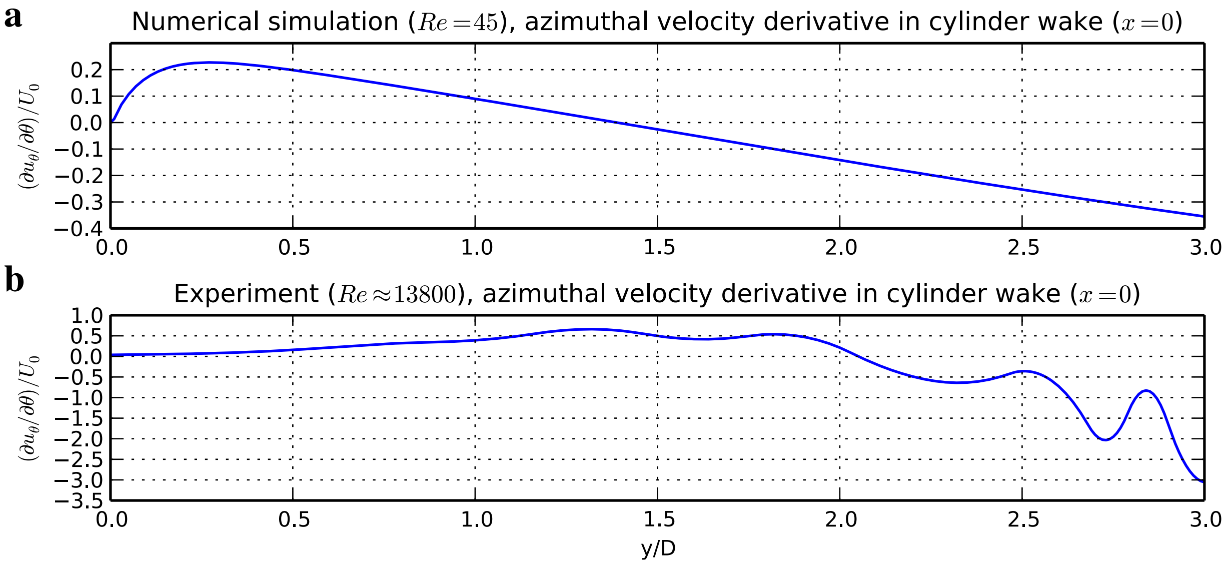

Supplementary Figure 8: Derivative of azimuthal velocity obtained from numerical and experimental investigations. a, Derivative of azimuthal velocity (at the center line) from numerical simulations of the flow around cylinder at $Re = 45$. **b,** Derivative of azimuthal velocity from experimental time averaged LDV measurements of the vertical velocity component in the wake behind cylinder in a soap film experiment, parameters $D = 6.9$ mm, $U_0 = 2.0$ m/s, and $Re \approx 13800$. The derivative of azimuthal velocity is obtained using cubic spline interpolation of velocity data.

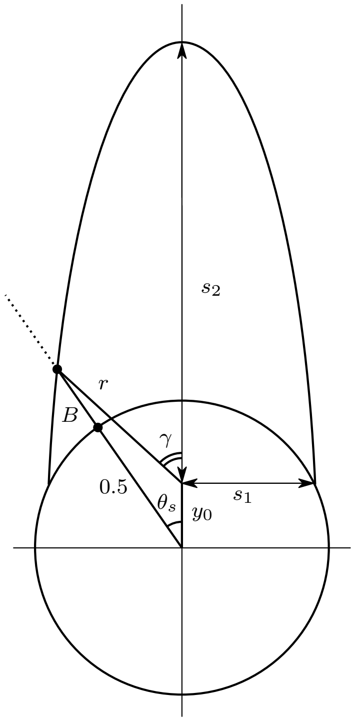

Supplementary Figure 9: Sketch of the geometrical problem for finding the equilibrium turn angle θ_s. The distance B from the cylinder surface to edge of the BFR is assumed to be known. The ellipse radius in polar coordinates r, the ellipse angle γ and the turn angle θ_s are the unknowns.

Supplementary Note 1: Solving for $\theta = \pm\theta_\mathrm{s}$

In this note, we show how to obtain the non-trivial solutions of Eq. (4) in the main paper and we show that these solutions are always stable. For convenience, the "hats" over dimensionless variables have been omitted. The angle θ_s can be obtained by solving a geometrical problem, where the length of the splitter plate (at unknown turn angle θ_s) inside the back flow region (BFR) is

$$B\left(\theta_\mathrm{s}\right) = \frac{1}{2}\left[\sqrt{\frac{4}{k+1}\left(L^2+L\right)+1}-1\right], \tag{1}$$

given that we know the total length of the splitter plate L and the coefficient k. A sketch of the geometrical problem is shown in Supplementary Fig. 9. At this point we assume that the shape of the back-flow region (BFR) is half of an ellipse (more information in Methods: estimating function $B\left(\theta\right)$) and its center is located at a distance y_0 from the center of the cylinder. The half ellipse is defined by semi-axes s_1 and s_2. The turn angle θ_s is defined as the angle between vertical center line of the ellipse (direction of the straight position) and the line going through the splitter plate.

Consider the point where the splitter plate intersects the BFR. Connecting the intersection point with the center of the ellipse yields a triangle (Supplementary Fig. 9). We are interested in the turn angle θ_s in the triangle, in which two sides are known. To complete the problem formulation, we introduce the ellipse radius r and angle γ. In total, we have three unknowns in our problem. We relate the ellipse radius and angle using ellipse equation in polar coordinates

$$r^2\frac{\sin^2\gamma}{s_1^2}+r^2\frac{\cos^2\gamma}{s_2^2}=1. \tag{2}$$

The second equation is sine rule in the constructed triangle

$$\frac{r}{\sin\theta_\mathrm{s}}=\frac{0.5+B}{\sin\left(\pi-\gamma\right)}=\frac{0.5+B}{\sin\gamma}, \tag{3}$$

where we have used the property of sine function $\sin\left(\pi-\gamma\right) = \sin\gamma$. Finally, the third and last equation is the cosine rule

$$r^2 = y_0^2+\left(0.5+B\right)^2-2y_0\left(0.5+B\right)\cos\theta_\mathrm{s}. \tag{4}$$

By using the sine rule (3), we may remove γ from the ellipse equation (2) and write

$$r^2s_1^2+\left(s_2^2-s_1^2\right)\sin^2\theta_\mathrm{s}\left(0.5+B\right)^2=s_1^2s_2^2. \tag{5}$$

Inserting the cosine rule (4) into expression (5), we arrive with a quadratic equation for the cosine of the turning angle, which has the solution

$$\cos(\theta_\mathrm{s}) = -\frac{b}{2a}\pm\frac{\sqrt{b^2-4ac}}{2a}, \tag{6}$$

where a, b and c are given by

$$\begin{aligned}
a &= \left(s_2^2-s_1^2\right)\left(0.5+B\right)^2,\\
b &= 2s_1^2y_0\left(0.5+B\right),\\
c &= s_1^2s_2^2-s_1^2y_0^2-s_2^2\left(0.5+B\right)^2.
\end{aligned}$$

Here auxiliary variables are $y_0 = 0.5\cos\theta_0$, $s_1 = 0.5\sin\theta_0$ and $s_2 = B_{\max} + 0.5 - y_0$. By analyzing the obtained roots of (6) with respect to the original problem, one finds that the root with plus sign corresponds to solution of the posed problem. Therefore the final expression for the turn angle is

$$\theta_{\mathrm{s}} = \arccos\left[-\frac{b}{2a} + \frac{\sqrt{b^2 - 4ac}}{2a}\right].$$

The explicit expression derived for θ_{s} corresponds to the blue lines in Figs. 1d, 3b and 5c in the main paper.

Next, we determine the stability of the skewed positions θ_{s} in a similar fashion as for $\theta = 0$ in the main text. Expanding the torque expression (4) in the main paper around $\theta = \theta_{\mathrm{s}}$ and using the fact that for the skewed solution $(1+k)\left[B^2(\theta_s) + B(\theta_s)\right] - L^2 - L = 0$, we arrive with the first-order term

$$\left.\frac{\partial T}{\partial \theta}\right|_{\theta=\theta_{\mathrm{s}}} = \sin(\theta_{\mathrm{s}}) \left.\frac{\partial\left\{(1+k)\left[B^2(\theta) + B(\theta)\right] - L^2 - L\right\}}{\partial \theta}\right|_{\theta=\theta_{\mathrm{s}}} A\rho_{\mathrm{f}} U^2 D^2.$$

First, note that $\sin(\theta_{\mathrm{s}}) > 0$ if $\theta_{\mathrm{s}} > 0$ (and $\sin(\theta_{\mathrm{s}}) < 0$ if $\theta_{\mathrm{s}} < 0$) for the range $-\theta_0 < \theta_{\mathrm{s}} < \theta_0$, where $\theta_0 \approx 55$ degrees (see Methods: estimating parameters $B_{\max}$ and k in main paper). Then, from the sketch in Supplementary Fig. 9, we find

$$\left.\frac{\partial B}{\partial \theta}\right|_{\theta>0} < 0 \quad \text{and} \quad \left.\frac{\partial B}{\partial \theta}\right|_{\theta<0} > 0,$$

which leads to

$$\left.\frac{\partial T}{\partial \theta}\right|_{\theta=\theta_{\mathrm{s}}} < 0.$$

This shows that the skewed solutions are always stable. Any deviation in positive angle direction will introduce a negative restoring torque and vice versa.

Supplementary Note 2: Obtaining drift direction from model

Here we explain how to obtain the direction of drift from theoretical model predictions. Inserting expression Eq. 7 in the main paper for $B(\theta_{\mathrm{s}})$ in Eq. (8) leads to a more explicit form of the drift angle

$$\alpha(\theta_{\mathrm{s}}) = \arctan\left(-\sin(\theta_{\mathrm{s}})\cos(\theta_{\mathrm{s}})\left[\sqrt{4(k+1)(L^2+L) + (k+1)^2} - (2L + [k+1])\right]\right).$$

Now we can determine the direction of the drift force F_{d}. By definition $k > 0$ and $L > 0$, therefore the expression in square brackets is always larger than zero. When $-\theta_0 \leq \theta_{\mathrm{s}} \leq \theta_0$ (here $\theta_0 \approx 55$ degrees, see Methods: estimating parameters $B_{\max}$ and k), the cosine is always positive and the sign of α is determined by $\sin(\theta_{\mathrm{s}})$ alone. In particular, if $\theta_{\mathrm{s}} > 0$ then $\alpha < 0$ (or vice versa), which means that the splitter plate is tilted in the same direction as the body drifts, in agreement with our experimental (Fig. 2) and numerical (Fig. 3 and Fig. 6) findings. Note that if α is negative, the object drifts to the left.
